# A Newly Identified Essential Complex, Dre2-Tah18, Controls Mitochondria Integrity and Cell Death after Oxidative Stress in Yeast

**DOI:** 10.1371/journal.pone.0004376

**Published:** 2009-02-05

**Authors:** Laurence Vernis, Céline Facca, Emmanuelle Delagoutte, Nicolas Soler, Roland Chanet, Bernard Guiard, Gérard Faye, Giuseppe Baldacci

**Affiliations:** 1 Régulation de la réplication de l'ADN des eucaryotes, UMR2027 CNRS/Institut Curie, INSERM, Université Paris-Sud, Orsay, France; 2 CNRS, Centre de Génétique Moléculaire, Gif-sur-Yvette, France; 3 Stabilité des génomes et stress génotoxiques, UMR2027 CNRS/Institut Curie, Orsay, France; University of Massachusetts Medical School, United States of America

## Abstract

A mutated allele of the essential gene *TAH18* was previously identified in our laboratory in a genetic screen for new proteins interacting with the DNA polymerase delta in yeast [1]. The present work shows that Tah18 plays a role in response to oxidative stress. After exposure to lethal doses of H_2_O_2_, GFP-Tah18 relocalizes to the mitochondria and controls mitochondria integrity and cell death. Dre2, an essential Fe/S cluster protein and homologue of human anti-apoptotic Ciapin1, was identified as a molecular partner of Tah18 in the absence of stress. Moreover, Ciapin1 is able to replace yeast Dre2 *in vivo* and physically interacts with Tah18. Our results are in favour of an oxidative stress-induced cell death in yeast that involves mitochondria and is controlled by the newly identified Dre2-Tah18 complex.

## Introduction

Apoptosis is a cell death program allowing homeostatic equilibrium in multicellular organisms. It is a key process to eliminate damaged cells during aging or following cellular injuries, or extra cells during developmental processes. It is a subject of debate whether such a form of cell death program exists in unicellular organisms, as there is no benefit for a single cell to kill itself. In nature, single cell organisms such as yeasts or bacteria develop in colonies or in biofilms and under those conditions, cells are in contact with each other. Several reports have shown that the death of some cells is of benefit to the rest of the colony and confers growth advantage over their competitors. In yeast colonies, an ammonia signal is emitted upon oxidative stress that triggers yeast death only in the centre of the colony [2]. Moreover this phenomenon is advantageous to younger cells at the periphery as physically removing central dying cells reduces growth at the outer margin of the colony. Also yeast cells containing killer viruses have been reported to induce genetically programmed cell death among non-infected cells in the population [3] allowing survival advantage for viruses containing *versus* non-containing cells in chronologically aged cultures.

From a molecular point of view there are indications that yeast cells in some circumstances die with biochemical markers of apoptosis. After exposure to hydrogen peroxide (H_2_O_2_) or acetic acid, yeast cells exhibit DNA strand breakage, chromatin fragmentation and exposure of phosphatidylserine on the outer plasma membrane [4]–[6]. These molecular changes associated with death require active protein synthesis since they are prevented by cycloheximide, a potent translation inhibitor [4]. Such apoptotic markers have also been described after exposure to other external stresses such as NaCl [7], [8], UV [9], high levels of the yeast mating pheromone alpha factor [10], or even in the absence of external stress, in a mutated *cdc48* context. *CDC48* encodes an ATPase involved in the retrotranslocation of ubiquitinated proteins into the cytosol for processing by the proteasome, and one particular *cdc48* mutant has been shown to die exhibiting typical apoptotic markers [11], [12].

In yeast, orthologues of key regulators of late mammalian apoptotic events have so far been identified. The yeast protease Yor197wp (Yca1) behaves very much like a mammalian caspase: it is cleaved in the presence of hydrogen peroxide thus gaining caspase activity *in vitro*, with H_2_O_2_-induced apoptosis either being abrogated or increased when *YCA1* is deleted or overexpressed respectively [13]. Also, an Apoptosis Inhibiting Factor (AIF) homologue in yeast, Ynr074cp, has been reported that translocates from the mitochondria to the nucleus and is able to degrade yeast nuclear DNA and plasmid DNA in a similar manner to mammalian AIF [14]. Finally, yeast Nma111p was found to be homologous to the mammalian mediator of apoptosis HtrA2/Omi [15] and the presence of apoptotic markers in dead yeast cells exposed to H_2_O_2_ is dependent on Nma111p [16].

Nevertheless, despite accumulating data showing apoptotic phenotypes in unicellular organisms and the presence of orthologues of some key apoptotic regulators, it is still unclear whether fully integrated cell death pathways exist in yeast. In particular, the early steps of a defined cell death program are unknown, even though the heterologous expression of active forms of proapoptotic mammalian Bax in yeast induces cell death with apoptotic phenotypes, and targets mitochondria [17], [18]. No Bax orthologue has been found in yeast. Interestingly, it was recently reported that, similarly to the human cohesin Rad21 that is involved in apoptosis [19], [20], the yeast homologue of hRad21, Mcd1, is cleaved by the caspase-like protease Esp1 after exposure to lethal doses of hydrogen peroxide [21]. Further, the C-terminal part of Mcd1 translocates from the nucleus to the mitochondria and cell death is enhanced under those conditions.

In this study we have investigated the role of the yet uncharacterized yeast protein Tah18 that was previously identified in our laboratory through a genetic screen for synthetic mutants with *pol3-13*, a thermosensitive allele of *POL3* which encodes the catalytic subunit of DNA polymerase delta [1]. We first generated thermosensitive *tah18* mutants that proved to be highly resistant to an acute exposure to H_2_O_2_. We then demonstrated that H_2_O_2_-induced cell death is dependent on Tah18 abundance in the cell. In the presence of H_2_O_2_, we observed GFP-Tah18 relocalization to the mitochondria, which was not detectable using fluorescent mutated versions of Tah18. Next, we showed that mitochondria in *tah18* mutants are protected against H_2_O_2_-induced damage, with cytochrome c more degraded in wild-type than in *tah18* cells. *DRE2* was then identified as genetically interacting with *TAH18*, as mutated alleles are synthetic lethal, and Dre2 overexpression can suppress *tah18* growth defects at 35°C. Dre2 was recently characterized as a Fe/S protein [22] whose human homologue, Ciapin1, is an anti-apoptotic protein [23]. Ciapin1 was found to suppress the *Δdre2* lethality and interact physically with Tah18 in the absence of oxidative stress. Finally, expressing Tah18 and Dre2 as a single fusion protein allowed viability. Moreover, the fusion protein did not delocalize to mitochondria and prevented cell death in the presence of H_2_O_2_. From this work we conclude that Tah18 plays a “pro-death” role in response to oxidative stress. After exposure to lethal doses of H_2_O_2_, Tah18 targets the mitochondria and promotes cell death in yeast and thus is a component of a cell death program although no connexion between Tah18 and cell death had been anticipated. As no Bax/Bcl2 homologues have been described so far in this organism, we discuss the significance of these findings with respect to yeast physiology.

## Results

### 1. Identification of temperature sensitive *tah18* mutants which are more resistant than wild-type to hydrogen peroxide but not to other DNA damaging agents

**Figure 1:**
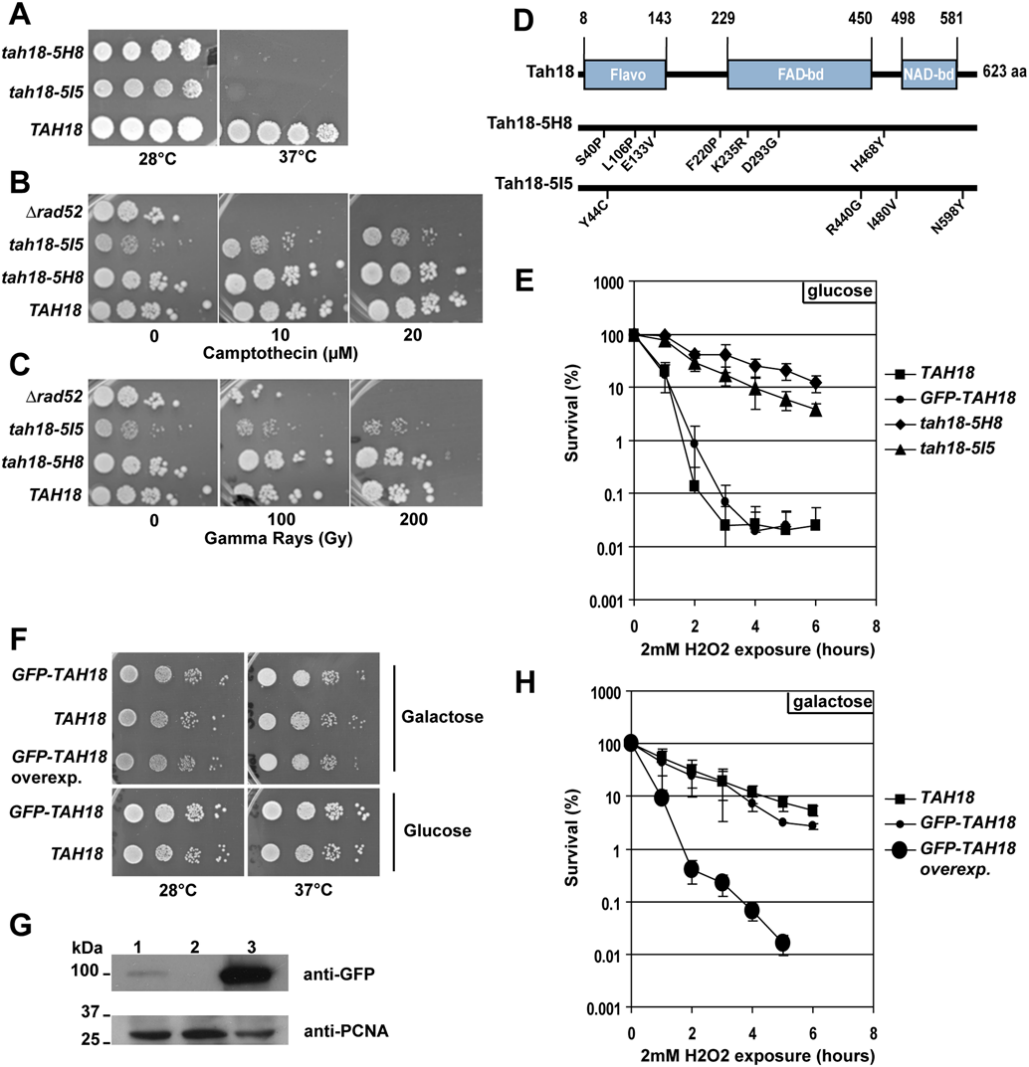
A–C. *Tah18-5H8* and *tah18*-*5I5* are thermosensitive mutants that are not involved in DNA repair after exposure to gamma rays or camptothecin. Ten-fold dilutions of exponentially grown yeast cells were spotted onto YPD medium (A–B) or YPD containing Camptothecin as indicated (C). Gamma rays irradiation was performed using a ^137^Cs with a dose rate of 0.850 Gy/s. Growth was assessed after 3 days at 28°C unless indicated. A *Δ rad52* mutant was used as a control. D. Tah18 exhibits three domains potentially involved in oxydo-reduction reactions. Flavodoxin, FAD-binding and NAD-binding domains were detected using Blast program. Mutations in *tah18*-*5H8* and *tah18*-*5I5* are indicated. E. *Tah18*-*5H8* and *tah18*-*5I5* are more resistant to H_2_O_2_ acute exposure than *TAH18*. Exponentially growing cells in YPD (1–5.10^6^ cells per milliliter of culture) were exposed to 2 mM H_2_O_2_ during 1 to 6 hours before being plated onto YPD. After 3 days at 28°C, the number of colony forming units (CFU) was counted and expressed as a percentage of the number of CFU at time zero to assess survival rate. Points are means of three independent experiments. Error bars are +/−standard error. F–G. Expressing GFP-TAH18 under the control of endogenous TAH18 promoter or overexpressing GFP-TAH18 under the control of the Gal1-10 promoter does not affect cell growth. F. Ten-fold dilutions of exponentially grown yeast cells from strains 11B3 expressing GFP-Tah18 under the control of endogenous TAH18 promoter (*GFP-TAH18*), 8C2 wild-type strain (*TAH18*) and 10H8 expressing GFP-Tah18 under the control of Gal1-10 promoter (*GFP-TAH18* overexp.) were spotted onto YPGal (inducing conditions for Gal1-10 driven expression) or YPD medium, and growth was assessed after 3 days at 28°C. G. Western blot analysis of GFP-Tah18 expression. Lane1: strain 11B3 expressing GFP-Tah18 under the control of endogenous *TAH18* promoter, Lane2: wild-type strain 8C2, Lane3: strain 10H8 expressing GFP-Tah18 under the control of Gal1-10 promoter. Protein extracts were prepared using the classical TCA method starting from yeast cultures grown in YPGal until a density close to 5.10^6^ cells per milliliter of culture. Equal amounts of total proteins were loaded on a 10% acrylamide gel and analysed by Western blot using anti-GFP antibodies. Anti-PCNA antibodies were used as loading control. H. Overexpressing *GFP-TAH18* renders cells more sensitive to H_2_O_2_. Exponentially growing cells in YPGal medium (1–5.10^6^ cells per milliliter of culture) were exposed to 2 mM H_2_O_2_ for 1 to 6 hours before being plated onto YPGal plates. After 3 days at 28°C, the number of colony forming units (CFU) was counted and expressed as a percentage of the number of CFU at time zero to assess survival rate. Points are the means of three independent experiments. Error bars are +/−standard error.

*TAH18* is an uncharacterized gene in yeast, which is essential for viability. A *tah18* mutant was originally identified in the laboratory through a genetic screen for synthetic mutants with *pol3-13*, a thermosensitive allele of *POL3* [1]. For studying *TAH18* in more detail we created a collection of thermosensitive *tah18* mutants (see Materials and Methods) using a previously described method based on mutagenic PCR [24]. On the basis of their growth capacity at 28°C and absence of growth at 37°C, two mutants (*tah18*-*5H8* and *tah18*-*5I5*) were selected for further characterization (Fig. 1-A). Sequencing analysis revealed that each mutant contains several mutations as shown in Fig. 1-D.

A previous study in yeast had also identified a *tah18* mutant through a genetic screen for conditional *tah* (*top1*T722A-hypersensitive) mutants exhibiting thermo-sensitive growth at 36°C in the presence of the camptothecin mimetic mutant *top1T722A* [25], [26]. Camptothecin (CPT) needs ongoing replication for specifically freezing the covalent TopoI-DNA complex and is thought to inhibit DNA replication *via* cytotoxic DNA lesions [27], [28]; alternatively the removal of CPT complexes requires repair enzymes. This original *tah18* mutant exhibited sensitivity to CPT, suggesting a role of the protein in DNA repair after drug treatment.

Thus, we tested the sensitivity of *tah18*-*5H8* and *tah18*-*5I5* mutants to chronic exposure to CPT as shown in figure 1-B. We also assessed the sensitivity of the two mutants to gamma ray irradiation which creates DNA damage, mainly in the form of base damage and double and single strand breaks [29]. Using drop tests, neither of the two mutants showed significant sensitivity to either gamma rays or to CPT compared to wild-type cells (Fig. 1-B–C). Thus, the two mutants are not impaired for DNA repair after exposure to CPT or gamma rays.

Blast analysis of the Tah18 sequence revealed the presence of three conserved domains suggesting redox activity (Fig. 1-D). The N-terminus includes a Flavodoxin-like domain that consists of about 170 residues with a flavin mononucleotide (FMN)-binding site. This domain is involved in electron transfer reactions, and has been found in various species. In the central and C-terminal part of the protein, blast analysis identified potential FAD- and NAD-binding domains also compatible with electron transfer activity. A search for proteins with the same domain organisation (Conserved Domain Architecture Retrieval Tool, CDART) revealed many potential oxydo-reductases involved in various processes, but no particular class emerged from this analysis.

We investigated the sensitivity of *tah18* mutants to agents that modify the redox balance of the medium. We used hydrogen peroxide (H_2_O_2_) to generate oxidative stress and tested acute exposure to lethal doses of this compound. Exponentially growing cells in complete glucose medium (YPD) were exposed to 2 mM H_2_O_2_ for up to 6 hours before being plated onto complete medium to assess their viability by counting the number of colony forming units (CFU). Both *tah18-5H8* and *tah18*-*5I5* mutants were highly resistant to H_2_O_2_ exposure as compared to wild-type from 2 hours as shown in Fig. 1-E. Similar results were obtained using 1mM or 3mM H_2_O_2_ (not shown). This might argue for Tah18 being involved in regulating intracellular H_2_O_2_ detoxification either negatively or positively, or/and in controlling cell death in the presence of H_2_O_2_.

### 2. Cell death after H_2_O_2_ exposure is dependent on the amount of Tah18

We then asked whether Tah18 overexpression affects sensitivity to H_2_O_2_. We used two strains expressing Tah18 with an N-terminal GFP tag, either under the control of the Gal1-10 promoter (overexpression in YPGal, strain 10H8, Table 1) or under the control of the endogenous *TAH18* promoter (endogenous expression, strain 11B3, Table 1). Both constructs are fully viable compared to the wild-type strain as shown by drop tests (Fig. 1-F), indicating that the GFP-Tah18 is functional and that overexpression of the GFP-Tah18 fusion protein is not toxic. Relative amounts of the fusion protein in both strains are shown in Fig. 1-G. When grown in galactose and exposed to 2 mM H_2_O_2_, the strain overexpressing GFP-Tah18 showed significantly higher sensitivity to H_2_O_2_ as compared to the control strain expressing GFP-Tah18 under native *TAH18* promoter or to the wild-type strain (Fig. 1-H). Thus, overexpressing the GFP-Tah18 protein renders the cells more sensitive to H_2_O_2_ in galactose medium.

**Table 1:** Strains table.

Strain	Genotype	Source
5C6	*MATa ura3 leu2 his3 dre2Δ::TRP1-CYH2^S^ pRS416-DRE2-URA3*	This study
5C8	*MATa ura3 leu2 his3 tah18Δ::TRP1-CYH2^S^ pRS416-TAH18-URA3*	This study
5H8	*MATa ura3 leu2 trp1 his3 tah18-5H8 (#31)*	This study
5I5	*MATa ura3 leu2 trp1 his3 tah18-5I5 (#43)*	This study
8C1	*MATa/alpha ura3/ura3 leu2/leu2 trp1/trp1*	Gérard Faye
8C2	*MATalpha ura3 leu2 trp1 lys2 cyh2^R^*	Gérard Faye
8C4	*MATa/alpha ura3/ura3 leu2/leu2 trp1/trp1 cyh2^R^/cyh2^R^*	Gérard Faye
8D1	*MATalpha ura3 his3 dre2Δ::TRP1-CYH2^S^ pRS315-hCiapin1-LEU2*	This study
9A2	*MATalpha ura3 his3 dre2D::TRP1-CYH2^S^ pRS425-hCiapin1-LEU2*	This study
9D9	*MATa ura3 leu2 trp1 dre2Δ::DRE2-GFP HIS3-Mx6*	This study
10G3	*MATa ura3 leu2 trp1 his3 dre2P221S*	This study
10H8	*MATa ura3 leu2 his3 TRP1-GAL1-GFP-TAH18*	This study
11B3	*MATa ura3 his3 tah18Δ::TRP1-CYH2^S^ pRS305-TAH18Promoter-GFP-TAH18-LEU2*	This study
12C2	*MATalpha ura3 leu2 his3 DRE2::TRP1-GAL-3HA-DRE2*	This study
12E3	*MATa ura3 his3 dre2Δ::TRP1-CYH2^S^ tah18Δ::TRP1-CYH2^S^ pRS315-LEU2-DRE2Promoter-DRE2-TAH18*	This study
12E6	*MATalpha; ade2-1; ura3-1; his3-11,15; trp1-1; leu2-3,112; can1-100; UBR1::HIS3-GAL-HA-UBR1* (background W303)	This study
12E8	*MATa his3 leu2::GFP-TAH18-pRS305-LEU2 tah18Δ::TRP1-CYH2^S^ DRE2::TRP1-GAL-3HA-DRE2*	This study
12F2	*MATa ura3 UBR1::HIS3-GAL-HA-UBR1 leu2::GFP-TAH18-pRS305-LEU2 tah18Δ::TRP1-CYH2^S^*	This study
EGY48	*MATa his3 trp1 ura3 LexAop-LEU2*	Clontech

All strains used in this study are congenic excepted EGY48 (Clontech) and 12E6 (W303).

It is noteworthy that the two control strains (wild-type 8C2 and GFP-Tah18 11B3) were more resistant to H_2_O_2_ when grown in galactose (Fig. 1-H) compared to glucose (Fig. 1-E). A previous report had indicated that yeast sensitivity to H_2_O_2_ is dependent on the carbon source since yeast cells grown in respirable glycerol-containing medium exhibit a higher resistance to H_2_O_2_ cell killing compared to yeast cells grown in fermentable glucose-containing medium [30]. This result can be explained by an increased expression of detoxification systems such as catalase, superoxide dismutase and glutathione peroxidases in respirable compared to fermentable carbon sources [31].

To summarize, we have shown that mutated Tah18 renders cells more resistant to H_2_O_2_ exposure, whereas overexpression of GFP-Tah18 renders cells more sensitive to the same treatment. This result indicates that cell death after H_2_O_2_ exposure is dependent on the amount of Tah18 and that *tah18-5H8* and *tah18-5I5* mutants are not *gain-of-function* mutants regarding their cell death phenotype in H_2_O_2._ Thus, Tah18 may negatively regulate intracellular H_2_O_2_ detoxification, and/or positively promote cell death.

### 3. GFP-Tah18 translocates to mitochondria upon H_2_O_2_ treatment but not GFP-Tah18 mutated versions

**Figure 2:**
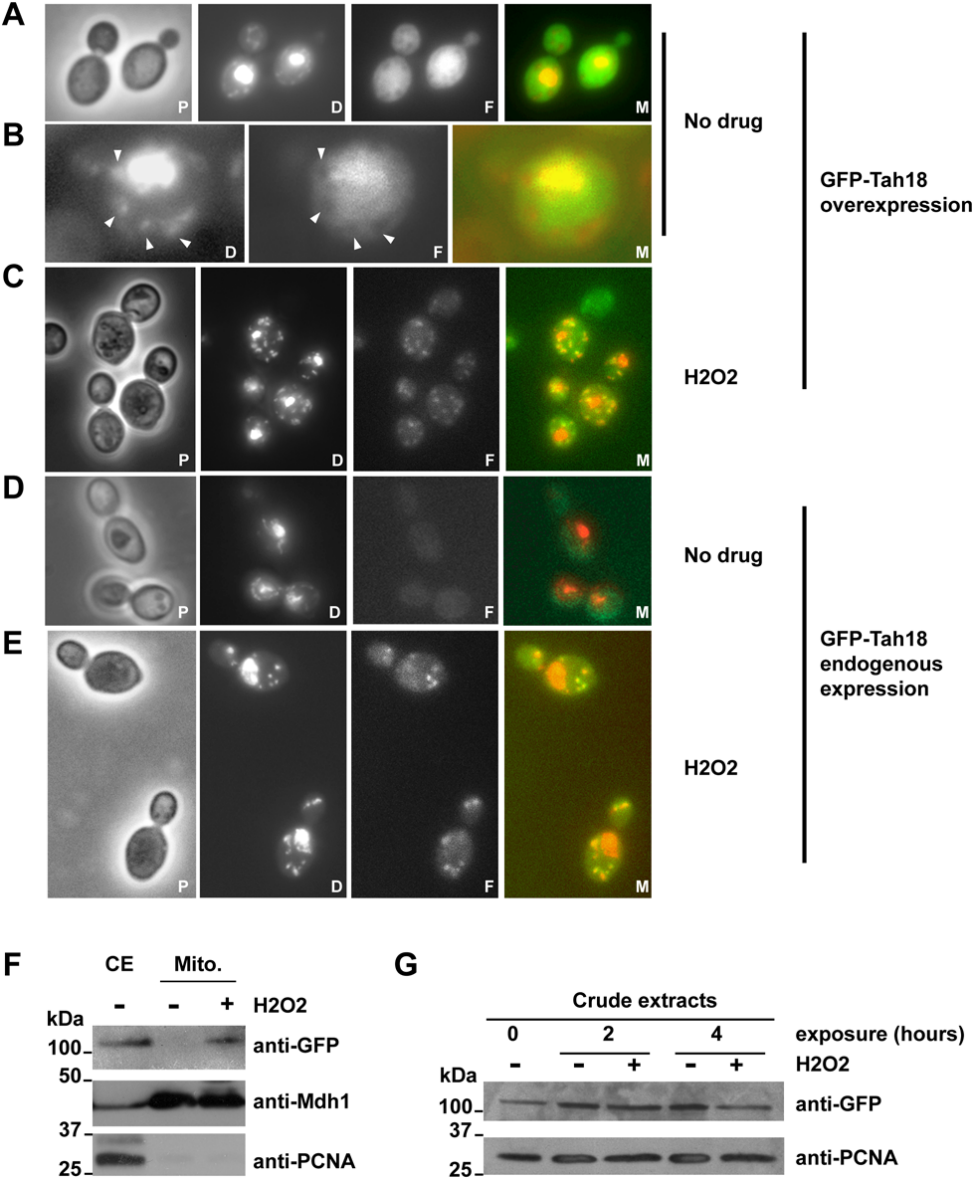
GFP-Tah18 relocalizes to the mitochondria after H_2_O_2_ exposure. A–E: Fluorescence microscopy of live cells expressing GFP-Tah18 under the control of the Gal promoter in an exponential culture in YPGal (strain 10H8, panels A–C) or under the control of endogenous *TAH18* promoter in YPD (strain 11B3, panels D–E). Panel B is a magnification of a single cell from A. Mitochondria are indicated with white arrows. Live cells were directly stained with DAPI at 0.05 mg/ml in the mounting solution to visualize nuclear and mitochondrial DNA. GFP fluorescence was detected using FITC filterset. When indicated, cells were treated with 2 mM H_2_O_2_ during 4 hours before direct observation. P: Phase contrast; D: DAPI; F: FITC, M: Merge. F. Western blot analysis of purified mitochondria. Crude extracts (CE) were prepared using classical TCA method starting from yeast cultures grown in YPD until a density close to 5.10^6^ cells/mL. Cells were treated with or without 2 mM H_2_O_2_ during 4 hours before processing. Mitochondrial extracts (Mitos.) were fractionated as described in the Materials and Methods section using sucrose gradients. 10 µg of each extract was loaded. Anti-Mdh1 antibodies were used as a loading control for mitochondrial extracts and anti-PCNA antibodies were used as a control for nucleo-cytosolic contamination of mitochondrial extracts. G. Western blot analysis of crude extracts. Crude extracts were prepared using classical TCA method starting from yeast cultures of strain 11B3 (expressing endogenous levels of GFP-Tah18) grown in YPD until a density close to 5.10^6^ cells/mL, and treated with or without 2 mM H_2_O_2_ during 2 and 4 hours before processing.

To gain a better insight into Tah18 function in yeast, we next investigated Tah18's intracellular localization. We used live cell fluorescent microscopy to examine the localization of the GFP-Tah18 fusion protein in the strain 10H8 (overexpression) and 11B3 (endogenous expression). When cells were grown exponentially in galactose, the GFP-Tah18 fluorescence pattern is diffuse in the cell, in contrast with the DAPI fluorescence pattern that specifically shows nucleus and mitochondria places (Fig. 2-A), suggesting that GFP-Tah18 is mainly cytosolic when overexpressed. Moreover, at location of DAPI-stained mitochondria we noticed the absence of any green fluorescence (black spots indicated by arrows) suggesting that GFP-Tah18 is absent from the mitochondria as shown on the magnification panel (Fig. 2-B).

After a 4 hour H_2_O_2_ exposure, the localization of the GFP-Tah18 fluorescence changed dramatically and revealed a mitochondrial pattern, as shown by colocalization with DAPI-stained mitochondria (Fig. 2-C). Thus, fluorescence microscopy suggests that GFP-Tah18 relocalizes to the mitochondria after exposure to H_2_O_2_.

Gal-controlled expression is very high in yeast and may provoke additional phenotypes due to protein overexpression even though cell viability in the absence of H_2_O_2_ in this strain is very similar to that seen in wild-type (Fig. 1-F). For this reason we next investigated the yeast strain expressing GFP-Tah18 under the control of the endogenous *TAH18* promoter (11B3). This strain exhibited no obvious phenotype compared to wild-type in exponentially growing cultures or after an acute exposure to H_2_O_2_. By Western blotting using an anti-GFP antibody, very low amounts of GFP-Tah18 protein were detected as compared to the strain 10H8 grown in galactose (Fig. 1-G). In accordance, we could not detect any fluorescence by microscopy when the cells were grown in the absence of H_2_O_2_ (Fig. 2-D). However, after 4 hours exposure to H_2_O_2_ 2 mM GFP-Tah18 also exhibited a mitochondrial distribution (Fig. 2-E) suggesting again that an acute exposure to H_2_O_2_ provokes Tah18 relocalization to the mitochondria when GFP-Tah18 is expressed under the native *TAH18* promoter.

To confirm the microscopy data, we used a cell fractionation approach. We isolated mitochondria from 11B3 cells exponentially grown in glucose medium in the presence or absence of H_2_O_2_ using sucrose density centrifugation as described previously [32]. The presence of the GFP-Tah18 fusion protein was assessed by Western blotting using an anti-GFP antibody. Results in figure 2-F show the absence of GFP-Tah18 in the mitochondria purified from untreated cells, whereas GFP-Tah18 is detected in mitochondria from cells exposed to H_2_O_2_.

Finally, Figure 2-G shows that GFP-Tah18 is maintained in a steady state in a crude extract after exposure to H_2_O_2_, arguing that GFP-Tah18 appearing in the mitochondria is not due to a general increase in GFP-Tah18 level induced by H_2_O_2_.

Taken together, these results show that GFP-Tah18 is localized outside the mitochondria in exponentially growing cells, and relocalizes to the mitochondria after exposure to H_2_O_2_.

In order to address whether *tah18-5H8* and *tah18-5I5* mutants showed an altered localization pattern after H_2_O_2_ exposure, GFP-tagged versions of both mutants were constructed. Growth capacity and resistance to H_2_O_2_ were similar in tagged or untagged *tah-5H8* and *tah18-5I5* versions (not shown). Using live cell fluorescence microscopy we were unable to detect GFP in the mitochondria up to 6 hours of exposure to H_2_O_2_, indicating that GFP-Tah18-5H8 and GFP-Tah18-5I5 are affected for delocalization to the mitochondria after oxidative stress (not shown).

### 4. Mitochondria integrity is preserved in *tah18* mutants after H_2_O_2_ exposure

**Figure 3:**
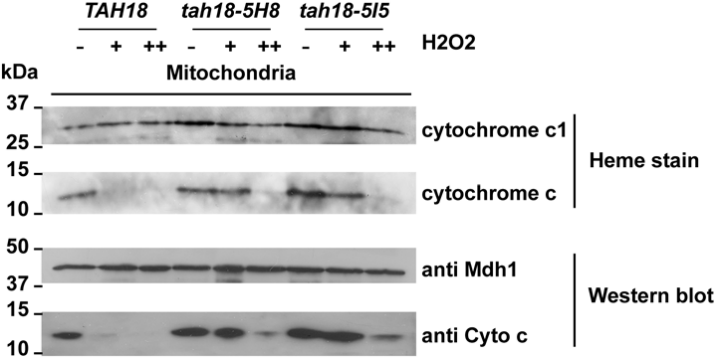
Mitochondria integrity during H_2_O_2_ stress is more preserved in *tah18* mutants than in wild-type *TAH18* cells. Heme staining and Western blot analysis of cytochrome c in purified mitochondria reveal that cytochrome c degradation is prevented in *tah18* mutants. Mitochondrial extracts were fractionated as described in the Materials and Methods section, starting from exponentially grown cells (5.10^6^ cells/ml or less) in YPD medium. The concentration of each extract was assessed using the Bradford assay and 10 µg was loaded per well. For heme staining of mitochondrial cytochromes c and c1 (respectively Cyc1 and Cyt1), samples were incubated with Laemmli buffer containing dithiothreitol 50 mM for 30 min at 4°C before migration on lithium dodecyl sulfate-PAGE and electro-transfer onto nitrocellulose membrane. Heme-associated peroxidase activity was revealed using ECL plus Western blotting detection system (GE Healthcare) directly on the nitrocellulose membrane. Anti-Mdh1 antibodies were used as loading control of mitochondrial extracts.

H_2_O_2_ exposure results in mammalian cell death that is accompanied by cytochrome c release from mitochondria to the cytosol before degradation [33]. In yeast, cytochrome c (Cyc1) release from mitochondria into the cytosol has also been shown to some extent after acetic acid treatment of cells [34]. We used heme staining to assess holo-cytochrome c status in the mitochondria [35]. Mitochondria were purified from cells treated with 1 mM or 2 mM H_2_O_2_ for 1 hour. As shown in figure 3-A, heme staining of purified mitochondria revealed that the holo-cytochrome c is still intact in mitochondria from *tah18-5H8* and *tah18-5I5* cells after 1 hour exposure to 1 mM H_2_O_2_ but not in mitochondria from wild-type cells. After 1 hour of exposure to 2 mM H_2_O_2_, holo-cytochrome c starts to be destabilized also in the mitochondria from *tah18* mutants. The mitochondrial holo-cytochrome c is thus more stable in mitochondria from *tah18* mutants than in those from wild-type cells. This result correlates with the higher survival rate of *tah18* mutants compared with wild-type cells after an acute exposure to H_2_O_2_.

Furthermore, Western blotting analysis using an anti-cytochrome c antibody showed that Cyc1 disappears from the mitochondrial extracts with a kinetics very similar to its loss of peroxidase activity (as detected by heme staining) (Fig. 3-A). We were also unable to detect Cyc1 in any of the cytosolic extracts from the same experiment (not shown) indicating that if Cyc1 is released from mitochondria into the cytosol after H_2_O_2_, it must be rapidly degraded.

### 5. *DRE2* is a multi copy suppressor of *tah18* thermosensitivity, while *dre2* and *tah18* mutants are synthetic lethal

In an attempt to identify Tah18 partners, we first tried to identify genetic interactions with *tah18* mutants. We transformed the *tah18-5I5* mutant strain with a multicopy 2μ-based yeast DNA library made in YEp13 vector (LEU2 marker). Transformants were selected on SC–LEU medium at the permissive temperature (28°C) and then replica plated onto complete medium at various restrictive temperatures ranging from 32°C to 37°C. Growing colonies were selected and plasmid DNA was recovered from *E*. *coli*. More than 250 plasmids were assessed by re-transformation of the mutant and selection for thermoresistance before analysis with restriction enzymes and sequencing.

When the colonies were grown at 37°C, 20 plasmids were recovered, all containing *TAH18*.

**Figure 4:**
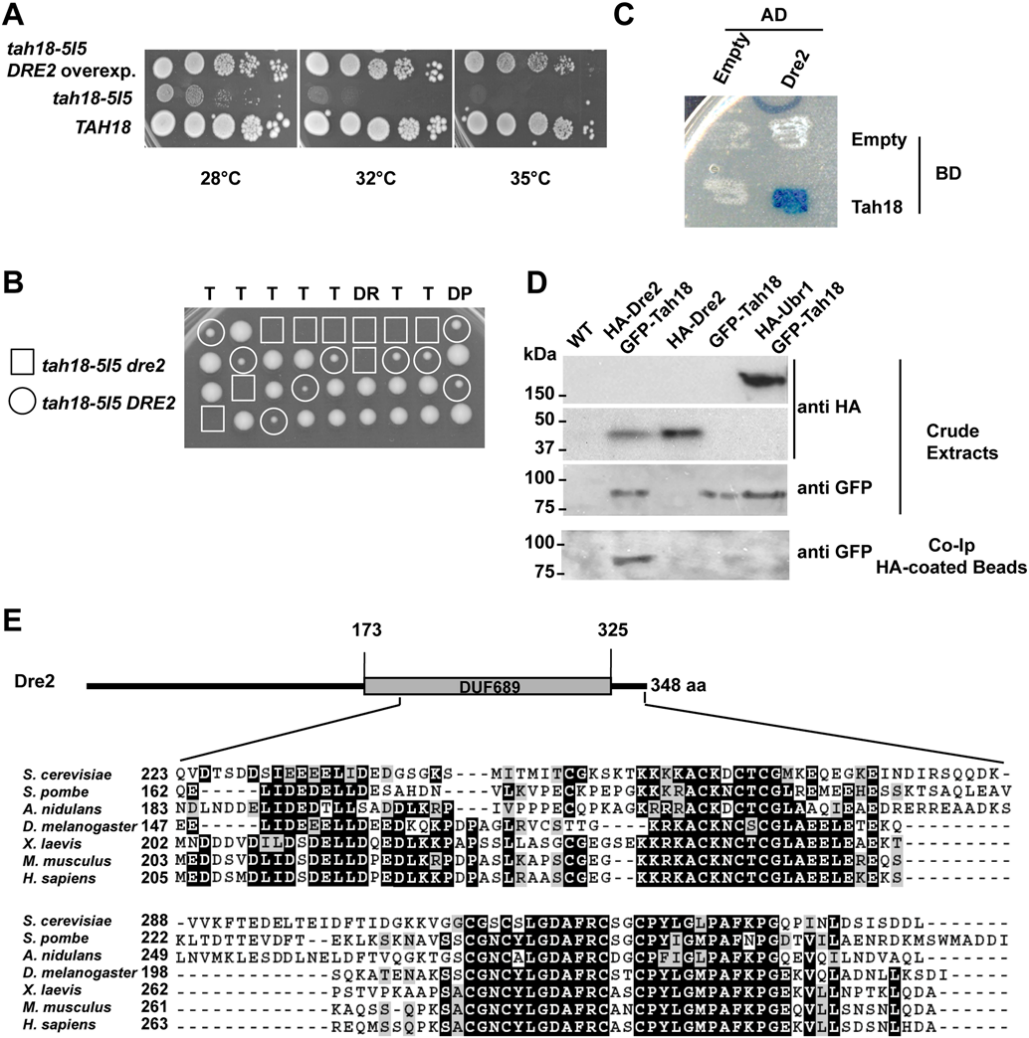
*DRE2* and *TAH18* interact genetically and their products interact physically *in vivo*. A. *DRE2* is a multicopy suppressor of *tah18*-*5H8* and *tah18*-*5I5*. Ten-fold dilutions of exponentially grown yeast cells were spotted onto YPD medium and growth was assessed after 3 days. When indicated, *DRE2* was expressed on a multicopy plasmid YEp13 in the *tah18*-*5I5* strain (two first lanes). B. *Dre2* and *tah18* mutants are synthetic lethal. Dissection plate from sporulated diploid *dre2-P221S/DRE2 tah18-5I5/TAH18* shows that spores harbouring both *dre2-P221S* and *tah18-5I5* mutated alleles are unable to grow. C–D. Dre2 and Tah18 interact physically *in vivo.* C. Two-hybrid was assayed according to manufacturer's instructions (see Materials and Methods). D. Crude extracts and co-IP HA coated beads were prepared as described in the M&M section. Anti-HA (Sigma) and anti-GFP (Roche) were used for Western blot analysis. Lane1: 8C2 WT, Lane2: 12E8 HA-Dre2 GFP-Tah18, Lane3: 12C2 HA-Dre2, Lane4: 11B3 GFP-Tah18, Lane5: HA-Ubr1 GFP-Tah18. E. Dre2 is a highly conserved protein. Alignments have been made using ClustalW and Boxshade at EMBnet. Identities are shaded in black and similarities are shaded in grey.

At 32°C or 35°C, in addition to 100 *TAH18*-containing plasmids, we recovered 142 plasmids all containing the same ORF, *YKR071C/DRE2*. No other ORF appeared to complement the *tah18* growth defect at both restrictive temperature 32°C or 35°C. Thus, *DRE2* overexpression is able to suppress *tah18* thermosensitivity at 35°C (see Fig. 4-A).

*DRE2* is an essential gene for vegetative growth but remained uncharacterized until a recent study showed that Dre2 is involved in cytosolic Fe/S protein biogenesis [22]. It is noteworthy that a *dre2* allele was previously identified in our laboratory that was synthetic lethal with *pol3-13*, a thermo-sensitive allele of DNA polymerase delta [1]. Sequencing that allele indicated that Phenylalanine 221 is substituted with Serine.

We next asked whether this *dre2-P221S* mutated allele and *tah18-5I5* allele exhibit a genetic interaction. *Tah18-5I5* and *dre2-P221S* haploid mutant strains (strains 5I5 and 10G3, Table 1) were crossed and diploids were selected. After sporulation, none of the spores harbouring both *dre2* and *tah18* mutated alleles were able to grow, whereas spores with a single mutation in either *TAH18* or *DRE2* were growing, thus showing that the combination of *tah18* and *dre2* alleles is synthetic lethal (see Fig. 4-B).

### 6. Tah18 protein interacts physically with Dre2 protein

We used co-immunoprecipitation to test whether Tah18 and Dre2 interact *in vivo*. As shown in figure 4-D, Hemagglutinin-tagged Dre2 (HA-Dre2) is able to specifically co-immunoprecipitate GFP-Tah18 indicating that HA-Dre2 and GFP-Tah18 interact in yeast extracts, and that this interaction is not mediated by the tags.

We then confirmed this interaction using the LexA-based two-hybrid technology (Clontech, see Materials and Methods). Full length proteins were expressed in this system as a fusion, either with the Binding domain (Tah18) or the Activating domain (Dre2). The expressed proteins showed a clear interaction, as indicated by growth and blue colour as compared with the controls on selective medium (Fig. 4-C).

### 7. Promoting Dre2-Tah18 interaction reduces H_2_O_2_-induced cell death and Tah18 localization to mitochondria

Since Dre2 interacts with Tah18, and overexpressing Dre2 can suppress *tah18* growth defect, we next tested whether Dre2 is also able to translocate to the mitochondria in the absence or presence of H_2_O_2_.

**Figure 5:**
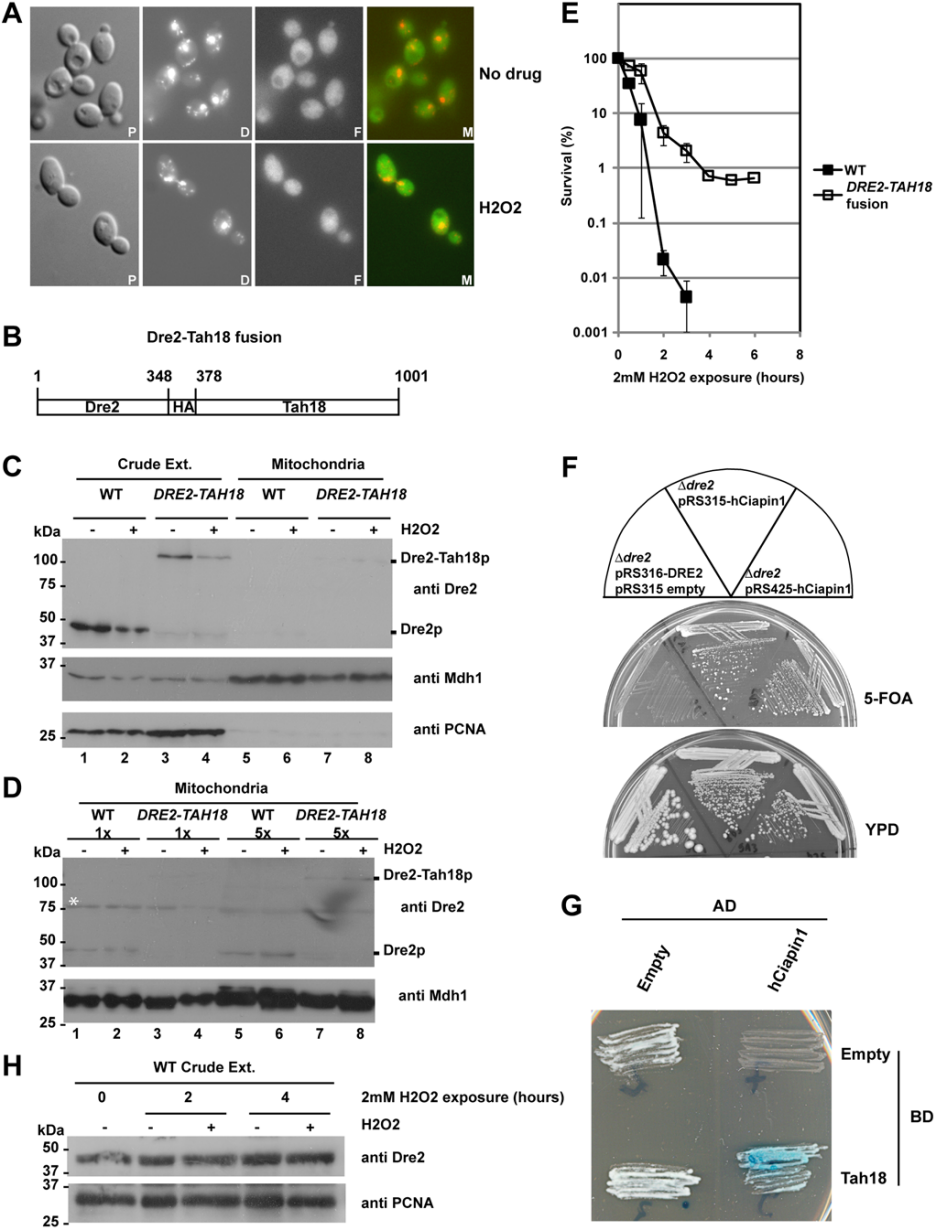
A–D. Neither Dre2p nor the Dre2-Tah18 fusion protein relocalize to mitochondria upon H_2_O_2_ treatment. A. Fluorescence microscopy of live cells expressing Dre2-GFP in an exponential culture in YPD (strain 9D9, panel A). Live cells were directly stained with DAPI at 0.05 mg/ml in the mounting solution to visualize nuclear and mitochondrial DNA. GFP fluorescence was detected using FITC filterset. When indicated, cells were treated with 2 mM H_2_O_2_ during 4 hours before direct observation. P: Phase contrast; D: DAPI; F: FITC, M: Merge. B. Dre2 and Tah18 were expressed as a fusion protein. C. Western blot analysis of crude and mitochondrial extracts using anti-Dre2 antibody. Crude extracts were prepared using classical TCA method starting from yeast cultures grown in YPD until a density close to 5.10^6^ cells/mL in the absence (−) or presence (+) of 2 mM H_2_O_2._. Mitochondrial extracts were fractionated as described in the Materials and Methods section using sucrose gradients from cells grown in the absence (−) or presence (+) of 2 mM H_2_O_2._ 10 µg of yeast crude extracts or 50 µg of mitochondria were loaded per well. WT: Wild-type strain, *DRE2-TAH18*: 12E3 strain expressing the fusion Dre2-Tah18. Anti-Mdh1 antibodies were used as a loading control for mitochondrial extracts and anti-PCNA antibodies were used as a control for nucleo-cytosolic contamination of mitochondrial extracts. D. Western blot analysis of increasing amounts of mitochondrial extracts. The white star shows a non specific band revealed by the anti-Dre2 antibody. 50 µg (1×) or 250 µg (5×) of mitochondrial extracts were loaded per well. E. Forcing Dre2-Tah18 interaction improves resistance to H_2_O_2_. Exponentially growing cells in YPD (1–5.10^6^ cells per milliliter of culture) were exposed to 2 mM H_2_O_2_ during 1 to 6 hours before being plated onto YPD. After 3 days at 28°C, the number of colony forming units (CFU) was counted and expressed as a percentage of the number of CFU at time zero to assess survival rate. Points are means of three independent experiments. Error bars are +/−standard error. F–G. Human Ciapin1 complements a *Δdre2* deletion and interacts *in vivo* with Tah18. F. The human Ciapin1 was expressed either on a URA3-containing centromeric (pRS315) or multicopy plasmid (pRS425) under the control of the TEF promoter. Growth was assessed after 3 days at 28°C, on 5-FOA or YPD plates. G. Two-hybrid was assayed according to manufacturer's instructions (see Materials and Methods). H. Dre2 is not degraded after exposure to H_2_O_2_. Western blot analysis of Dre2 in crude extracts after exposure to H_2_O_2_. Crude extracts were prepared using classical TCA method starting from yeast cultures grown in YPD until a density close to 5.10^6^ cells/mL in the absence (−) or presence (+) of 2 mM H_2_O_2_, during 2 or 4 hours. 10 µg of yeast crude extracts were loaded per well. Anti-PCNA antibody was used as a loading control.

We used live cell fluorescent microscopy to examine the localization of the Dre2-GFP fusion protein expressed under the native *DRE2* promoter (strain 9D9 Table 1). In the absence of stress, the Dre2-GFP fusion protein showed a diffuse cellular pattern that is in accordance with previously described cytosolic Dre2-GFP localisation [36]. No obvious change in Dre2-GFP distribution was observed after exposure to H_2_O_2_ (Fig. 5A), indicating that no massive Dre2 relocalization to the mitochondria occurred.

We then used an anti-Dre2 antibody (see Materials and Methods) that was raised against the Dre2 1-133 N-terminus part. Dre2 was detected as a single band with an expected size of 48-kDa in whole cell crude extract (Fig. 5-C lanes 1 and 2, Fig. 5-H) and was not found to be degraded or increased after a 2 or a 4 hours exposure to H_2_O_2_ (Fig 5-H). Very small amounts of Dre2 were also detected in mitochondrial extracts from wild-type cells in the absence or presence of H_2_O_2_ (Fig. 5-C lanes 5 and 6). Figure 5-D shows an overexposure of a Western blot analysis using the anti-Dre2 antibody of increasing amounts of mitochondrial extracts, indicating that a small fraction of Dre2 might be localized in mitochondria as previously described using a HA-tagged Dre2 [22] and that mitochondrial Dre2 amounts do not increase after exposure to H_2_O_2_ (Fig. 5-D lanes 1, 2, 5 and 6). This is consistent with the fact that cytosolic Dre2, unlike Tah18, does not relocalize to the mitochondria after oxidative stress.

It is possible that the Dre2-Tah18 interaction might affect Tah18 localization to the mitochondria after oxidative stress. We therefore tested whether Dre2 overexpression might improve resistance to H_2_O_2_. Dre2 was expressed under the control of the Gal1-10 promoter (strain 12C2, Table 1), and cells were exposed to 2 mM H_2_O_2_ for up to 6 hours as previously described. Resistance was indeed improved slightly when overexpressing Dre2 (not shown), indicating that stabilizing Dre2-Tah18 complex might prevent H_2_O_2_-induced cell death.

We next created a permanent interaction between Tah18 and Dre2 by fusing *DRE2* in frame with *TAH18*, in a strain deleted both for endogenous *DRE2* and *TAH18* (strain 12E3, Table 1 and Fig. 5-B). Surprisingly, this strain was fully viable, suggesting that the dissociation of Dre2 from Tah18 in wild-type cells might not be essential for viability. Immunodetection using an anti-Dre2 antibody in crude extracts indicated that the fusion protein is detected as a single band with an expected size of 110 kDa and behaves like Dre2 in the absence or presence of H_2_O_2_, as a small amount of the fusion protein is detected in the mitochondria in both conditions and does not relocalise to the mitochondria (Fig. 5-C lanes 3, 4, 7 and 8, Fig. 5-D lanes 3, 4, 7 and 8). Furthermore, when assaying for sensitivity to H_2_O_2_, the *DRE2-TAH18* fusion expressing strain was significantly more resistant than wild-type (as shown in figure 5-E). This result suggests that forcing the interaction between Dre2 and Tah18 *in vivo* prevents Tah18 relocalization to the mitochondria and decreases H_2_O_2_-induced cell death.

### 8. Human anti-apoptotic Ciapin1 is able to complement a *dre2* deletion and interacts with Tah18

Database searching for Dre2 homologues gave no significant hits with any other yeast protein, but revealed a high level of conservation among species in the C-terminus portion of the protein. This similarity was shared by 46 proteins in the Interpro database of protein families, and defines the entry signature DUF689 as shown in figure 4-E. The human homologue of Dre2p appeared to be the Ciapin1 (Cytokine Induced Apoptosis Inhibitor 1) protein, initially described as an inhibitor of apoptosis whose expression is regulated by growth factors and which is not related to Bcl2 or caspases [23]. We next investigated whether human Ciapin1 can complement the absence of Dre2 protein in yeast. Ciapin1 was subcloned either on a centromeric (pRS315, strain 8D1 Table 1) or multicopy (pRS425, strain 9A2 Table 1) vector under the control of the TEF promoter (promoter from the gene encoding translation elongation factor 1 alpha [37]). Both constructs were found to confer growth capacity in the absence of *DRE2* containing plasmid pRS316 (Fig. 5-F). This result is in accordance with recent work [22].

We then asked whether hCiapin1 interacts with yeast Tah18. As previously described, we used two-hybrid technology with both full length proteins. Like Dre2 (Fig. 4), hCiapin1 is able to interact physically with yeast Tah18 (Fig. 5-G).

We conclude that hCiapin1 and Dre2 share at least one common function, which is essential in yeast and might be dependent on the interaction with Tah18.

## Discussion

In this study we evidenced a previously uncharacterized protein complex, Dre2-Tah18, which controls yeast cells death in response to high doses of hydrogen peroxide. In the absence of exogenous oxidative stress, Tah18 and Dre2 physically interact outside the mitochondria. In the presence of oxidative stress, Tah18 is targeted to the mitochondria and controls mitochondrial integrity and cell death. Together, these results argue for the existence of a mitochondria-dependent cell death program in yeast involving Tah18.

### Tah18 targets mitochondria and is a pro-death factor in the presence of oxidative stress

We observed relocalization of the GFP-Tah18 fusion to mitochondria after exposure to high doses of H_2_O_2._ This relocalization is affected in *tah18-5H8* and *tah18-5I5* mutants. Moreover, we show that cytochrome c is rapidly destabilized and degraded in a Tah18-dependent manner under the same conditions. These findings under oxidative stress conditions are partly reminiscent of the properties of Bax in mammalian cells. Bax is a pro-apoptotic protein from the evolutionary conserved Bcl2 family, whose members regulate apoptosis either positively or negatively. Bax is inserted at the outer membrane of mitochondria at the early stage of apoptosis [38], [39]. This provokes mitochondrial membrane leakage and release of mitochondrial factors in the cytosolic compartment (*e. g.* AIF, cytochrome c, endoG…), which are in turn responsible for caspases activation, DNA degradation, and cell death.

Blast analysis reveals no significant sequence similarity between Bax and any yeast protein, and no Bax functional homologue has been characterized to date in yeast. Heterologous expression of human Bax in yeast is able to induce efficient mitochondria targeting and cell death only when the Bax conformation is stably changed due to mutations in the coding sequence or in the presence of a C-terminal tag [17], [18]. We show here that Tah18 galactose-driven overexpression induces neither Tah18 targeting to mitochondria nor toxicity in yeast in the absence of H_2_O_2_. Thus, Tah18 targeting to the mitochondria is accurately controlled by the redox balance. We artificially targeted GFP-Tah18 to the mitochondrial matrix using a Mitochondrial Localization Sequence (MLS) in the absence of oxidative stress. This strain was found to be viable in the presence of wild-type Tah18 and exhibited a strong mitochondrial GFP fluorescence (not shown), showing that Tah18 is not toxic when located in the mitochondria in the absence of oxidative stress, and suggesting that Tah18 may need to be modified by oxidative stress to relocalize to the mitochondria and be toxic, similarly to Bax.

From a molecular point of view, the signal that triggers Tah18 relocalization to the mitochondria is unknown and prediction software did not detect any Mitochondrial Localization Sequence in Tah18. Different mechanisms have been described that lead to non-resident protein translocation into the mitochondria after stress. In the case of the human CDK11 p110 isoform [40], human Bid (another pro-apoptotic Bcl2 family member) [41], [42], or yeast Mcd1 [21], protein cleavage is responsible for mitochondrial translocation. It is unlikely that Tah18 generates a cleavage product under oxidative stress, as the size of the GFP-Tah18 detected by Western blotting in mitochondrial extracts after H_2_O_2_ exposure is that of a full length protein. Translocation to the mitochondria after release from sequestration by a partner has been described for Bax [43]. To our knowledge, no such mechanism has been characterized to date with yeast proteins. We identified *DRE2*, which interacts genetically with *TAH18* in the absence of oxidative stress, and showed that gene products physically interact *in vivo*. Forcing the Dre2-Tah18 interaction by expressing them as a fusion results in an increased resistance to H_2_O_2_-induced cell death, and the fusion protein is not relocalized to the mitochondria. Also overexpressing Dre2 in yeast renders the cells more resistant to H_2_O_2_ arguing again that promoting the interaction between Tah18 and Dre2 helps prevent H_2_O_2_-induced cell death and that Dre2 might be considered as an “anti-death” factor. This view is reinforced by the fact that Ciapin1 has been identified as an anti-apoptotic factor in human cells [23], and is able to replace Dre2 efficiently in yeast and to interact with Tah18.

As described recently, Dre2 is an essential Fe/S cluster protein, containing both [2Fe-2S] and [4Fe-4S] clusters and involved in Fe/S cluster biogenesis [22]. It has been shown in bacteria that DNA binding and dimerization of the Fe/S-containing FNR protein from *Escherichia coli* are regulated by oxidation [44]. More generally, some particular Fe/S clusters, such as [4Fe-4S] can be seen as oxygen-responsive molecular switches as they are very sensitive to oxygen and ROS and are unstable under oxidative conditions [45], [46]. A likely hypothesis is that Dre2 is destabilized in the presence of H_2_O_2_, which might in turn affect Dre2-Tah18 complex stability.

Thus, we suggest that Tah18's pro-death function in yeast is promoted by oxidative stress both by destabilizing its interaction with Dre2 and allowing its efficient targeting to the mitochondria.

*Tah18* mutants are more resistant than wild-type to H_2_O_2_. Interestingly, yeast pretreated with low doses of H_2_O_2_ show an increased resistance to a subsequent higher concentration of hydrogen peroxide [47] due to the activation of detoxification genes that are controlled by the transcription factor Yap1 [48]. Mutants defective for adaptation to hydrogen peroxide were selected and some of them exhibited a constitutive sensitivity to chronic H_2_O_2_ but higher resistance to acute H_2_O_2_ probably due to unbalanced NADPH production, and overproduction of reduced glutathione [49]. It is a possibility that *tah18-5H8* and *tah18-5I5* mutants' redox balance might be affected even though, unlike the previously described mutants by Ng *et al.* [49], they are not sensitive to chronic H_2_O_2_ exposure (unpublished data). Increased detoxification of hydrogen peroxide could stabilize Dre2-Tah18 interaction in *tah18* mutants and thus avoid Tah18 relocalization to the mitochondria, as observed using fluorescent mutated Tah18 versions.

We also investigated whether Tah18's pro-death function could be revealed using other kinds of stresses. No GFP-Tah18 relocalization to the mitochondria was observed after gamma- or UV irradiation, or camptothecin treatment (not shown). Also *pol3-13* mutants shifted to the non-permissive temperature (37°C) for 4 hours showed no GFP-Tah18 relocalization, whereas under these conditions, more than 80% of the *pol3-13* cells die, arguing that Tah18 pro-death function is not involved in the death of cells exposed to gamma rays, UV, CPT or of *pol3-13* cells (not shown).

It would now be very informative to identify the molecular mechanisms leading to Tah18 relocalization to the mitochondria after exposure to H_2_O_2_, which might be affected in *tah18* mutants.

### What is the essential Dre2-Tah18 function in the absence of oxidative stress?

The Tah18 essential function is affected in the two mutants we used in this study *tah18*-*5H8* and *tah18*-*5I5*, as they are unable to grow at 37°C. This function is necessary in the case of impaired DNA replication as both mutants are synthetic lethal with *pol3-13* (unpublished data). As mentioned above, another *tah18* mutant was identified in a genetic screen for sensitivity to a mutated allele of *TOPI*, *top1T722A*, mimicking the cytotoxic action of camptothecin [25], [50] that might indicate a role for Tah18 in DNA repair/DNA integrity check. *Tah18*-*5H8* and *tah18*-*5I5* mutants are not sensitive to CPT at the permissive temperature (this work Fig. 1-B). Still, it is possible that the same function of Tah18 is required to overcome the stress generated both in the presence of *top1T722A* at 35°C and in *pol3-13* mutants. This function is compatible with an essential redox activity as suggested by the functional blocks identified in Tah18 (Fig. 1-D) but remains to be identified.

The Tah18-interacting protein Dre2 was reported to be involved in cytosolic Fe/S protein biogenesis [22]. Interestingly, the authors suggest a possible role for Dre2 in iron binding and iron reduction that would need a source of electrons for reductase activity. It is tempting to speculate that Tah18 is able to provide electrons for this reaction, as we have shown that Tah18 and Dre2 interact *in vivo*. Therefore, it is possible that the Dre2-Tah18 complex might be involved in iron reduction. This also raises the possibility that the release of bound iron from Dre2 might be affected by its interaction with Tah18, resulting in an increased production of hydroxyl radicals *via* the Fenton reaction. A separate study made a link between DNA replication and Fe/S clusters [51], and we also found a genetic link between *POL3* and *NBP35*, another Fe/S cluster protein also involved in Fe/S proteins biogenesis in the cytosol [1], [52]. Still, the exact relationship between Dre2-Tah18 and DNA metabolism is largely unknown despite preliminary evidence [1], [25], [26], [53] and has to be further explored.

In summary, this study shows that Tah18 has deleterious effects on mitochondria in the presence of lethal doses of H_2_O_2_, and promotes cell death. The Dre2-Tah18 complex might be seen as a molecular sensor for high oxidative stress levels that accurately controls an oxidative induced cell death pathway in yeast.

## Materials and Methods

Sequence analysis were performed at the ncbi server (http://www.ncbi.nlm.nih.gov/), search for potential mitochondrial targeting sequence was performed using TOMPRED (http://ihg.gsf.de/ihg/mitoprot.html).

### Strains and media

All yeast strains are congenic, excepted EGY48 (Clontech) and 12E6 (W303).

The *TRP1-CYH2^S^* disruption cassette has been described previously [24].

*Tah18-5H8* and *tah18-5I5* strains 5H8 and 5I5: The *TAH18* gene was amplified by PCR under mutagenic conditions as described previously [54]. The amplified *TAH18* DNA fragments were then integrated into the yeast genome at the *TAH18* locus by transformation of the 5C8 strain and selection for cycloheximide-resistant transformants at 28°C as described previously [24]. Transformants were then replica plated to tryptophan minus (trp-) plates to verify the full cassette replacement, and to plates containing 5-fluoroorotic acid to counter select the pRS416*-URA3-TAH18* plasmid. Temperature-sensitive mutants were selected by replicating to complete medium plates at 28°C and to 37°C.

*Dre2P221S* strain: mutated allele *dre2P221S* was amplified by PCR from original strain [1] using oligos 85 and 86, and was transformed directly in strain 5C6 after CycloR selection and *DRE2* plasmid shuffling on 5-FOA containing plates. Mutated *dre2* allele was sequenced for control.

Strain 9D9: PCR product using oligos 162 and 163 (see Table 2) onto pFA6a-GFP(S65T)-His3MX6 vector [55] was used to create a DRE2-GFP fusion; expression was verified by Western blot using an anti-GFP antibody (Roche).

**Table 2:** Oligos table.

Oligo	Sequence
85	GCATATACACTGTAAGTG
86	CCAATTGACGTCATTTAC
160	CGGACTAGTATGGCAGATTTTGGGATCTC
161	ATAAGAATGCGGCCGCCTAGGCATCATGAAGATTG
162	GGTCTTCCTGCTTTCAAGCCTGGTCAACCTATCAATTTGGACAGCATTTCAGATGACTTGCGGATCCCCGGGTTAATTAA
163	AAATTCACCTTCACCAAAGTAGACCAATTGACGTCATTTACTGAAACGAATGTGCAGGGTGAATTCGAGCTCGTTTAAAC
166	CCGCTCGAGCTGGAAAAATACTCATTAGGC
169	GCTCTAGAACTACGGTAAGCGTAACCACGACCAGGACCTTGACCTTGACCTTGACCCTCACCTTTGTATAGTTCATCCATGCC
172	CCGCTCGAGGTCGATAGCTTTACGCATCCG
173	AACTGCAGGCAGCGATTAATGGAAACACAG
174	AACTGCAGATGAGTAAAGGAGAAGAACTTTTCACTGG
176	GCTCTAGATCATCGAGCAAGAAAATCGTCATCC
177	ATAAGAATGCGGCCGCCTACCAAGTTTCCTGGATATATC
214	CGGAATTCCGGATGTCACAATACAAAACTG
215	CCGCTCGAGCGGTTACAAGTCATCTGAAATG
216	CGGAATTCCGGATGTCATCGAGCAAGAAAATCG
217	CCGCTCGAGCGGCTACCAAGTTTCCTGGATATATC
233	ATGACTACATAAAAAAAAAAAATTCGAAAAATTTCATGAGCCTTATTAACGAATTCGAGCTCGTTTAAAC
234	GCGTTACCTGTCTCCGATCCATAGAGGATGACGATTTTCTTGCTCGATGACATTTTGTATAGTTCATCCATGC
235	GATTAGTAATCATTTAATTTCATGACATATTAAAAAGGTATTCTAAGCAGGAATTCGAGCTCGTTTAAAC
236	GTAGTCACCGCCGGATGTATTAAAAGTAAACCAGTTTTGTATTGTGACATTTTGTATAGTTCATCCATGC
269	CGCCTGCAGCAAGTCATCTGAAATGCTGTCC
278	CGGCTGCAGTACCCATACGATGTTCCAGATTACGCTGGTGAGGGTCAAGGTCAAGGTCAAGGTCCTGGTCGTGGTTACGCTTACCGTAGTATGTCATCGAGCAAGAAAATCG
279	ATAAGAATGCGGCCGCCTACCAAGTTTCCTGGATATATC
313	CGGAATTCCGGATGGCAGATTTTGGGATCTC
314	CCGCTCGAGCGGCTAGGCATCATGAAGATTGC

Strain 10H8: PCR product using oligos 233 and 234 (see Table 2) onto pFA6a-*TRP1-*PGAL1-10*-GFP* [55] was used to replace endogenous *TAH18* promoter. GFP-Tah18 expression was verified by Western blot using an anti-GFP antibody (Roche).

Strain 12C2: PCR product using oligos 235 and 236 (see Table 2) onto pFA6a-*TRP1-*PGAL1-10*-*HA [55] was used to replace endogenous *TAH18* promoter. HA-Dre2 expression was verified by Western blot using an anti-HA antibody (Roche).

Strain 12E3: PCR product containing *DRE2* promoter plus *DRE2* gene was made using oligos 166 and 269 (see Table 2) and subcloned into replicative pRS415 vector using *Xho*I*-Pst*I restriction sites. PCR product containing *HA-TAH18* using oligos 278 and 279 (see Table 2) was subcloned into previous vector using *Pst*I*-Not*I restriction sites to obtain pRS415-*DRE2TAH18.* 5C6 strain was transformed with this latter plasmid and pRS416-*DRE2-URA3* plasmid was chased onto 5-FOA medium, before being crossed with 5C8 strain. pRS416-*TAH18-URA3* was chased from the diploid strain before sporulation, and a *Δtah18*:: *TRP1-CYH2^S^ dre2Δ*:: *TRP1-CYH2^S^*pRS415-*DRE2TAH18* spore was selected. Dre2Tah18 expression was verified by Western blot using an anti-HA antibody (Roche).

Strain 12E8 was obtained by crossing strains 12C2 and 11B3.

Strain 12F2 was obtained by crossing strains 12E6 and 11B3.

Yeast strains were grown in rich YP medium (1% yeast extract, Difco, 2% bacto peptone, Difco) supplemented with glucose (2%, YPD) or galactose (2%, YPGal) or in minimum medium YNB (0.67% yeast nitrogen base, Difco) supplemented with appropriate amino acids (40 mg/ml each) and glucose (2%) or galactose (2%). Agar (2%) (Difco) was added for growth on plates. H_2_O_2_ and CPT were purchased from Sigma.

### Cloning

All vectors used in this study have been described [55], [56].

Human Ciapin1 ORF was amplified using oligos 160 and 161 (see Table 2) using a human cDNA library made from Hela cells (kind gift from Olga Grigorieva) and subcloned into pRS315-Tef1 and pRS425-Tef1 promoter using *SpeI-NotI*.

PRS305-Prom*TAH18-GFP-TAH18* was constructed by amplifying independently the *TAH18* promoter region, the GFP ORF, and the *TAH18* ORF by PCR. The *TAH18* promoter region was amplified using oligos 172 and 173 (see Table 2) and cloned into pRS305 using *Xho*I and *Pst*I sites. The *TAH18* ORF was amplified using oligos 176 and 177 and cloned into the previous vector using *Xba*I and *Not*I sites. The GFP gene was amplified using oligos 174 and 169 onto pFA6a-GFP(S65T)-His3MX6 vector [55], the PCR product was restricted by *Pst*I and *Xba*I and subcloned into the previous vector. The resulting vector was linearized in the *TAH18* promoter using *Spe*I and integrated at the *TAH18* locus in the *Δtah18*:: *TRP1-CYH2^S^* pRS416-*TAH18* strain 5C8. The *URA3*-plasmid containing wild-type *TAH18* was then shuffled onto 5-FOA plates and the strain was verified by southern blot. GFP-Tah18 expression was verified by Western blot using a monoclonal anti-GFP antibody (Roche).

Multicopy 2μ-based yeast DNA library made in YEp13 vector (LEU2 marker) was a kind gift from B. Daignan-Fornier.

### Western blotting

Protein extracts were prepared using classical TCA methods [57]. Proteins were transferred from 10% acrylamide gels onto ECL membrane according to the manufacturer's instructions (Amersham). The membrane was blocked with 5% dried milk in Tris buffered saline containing 0.1% Tween 20. Proteins were detected using various antibodies (anti-GFP 1/1000 (Roche), anti-Cyc1 and anti-Mdh1 (kind gifts from Delphine Bernard and from Pfanner lab respectively). Anti-PCNA serum and Anti-Dre2 sera were raised in rabbit (Agrobio company) using 6×His-tagged PCNA and 6×His-tagged Dre2 1-133 N-terminus overexpressed in *E. coli* and purified on a nickel column (Qiagen). Primary antibodies were detected with secondary horseradish peroxidase-conjugated antibody (Jackson). The product of the reaction was detected with enhanced chemiluminescence (Amersham) according to the manufacturer's instructions.

### Protein extracts

Mitochondria were purified using sucrose gradients as previously described [32], except that DTT-containing wash buffer was not used.

For co-immunoprecipitation experiments, cells were grown exponentially in YPD and washed with PBS buffer before glass beads lysis in co-IP buffer (50 mM HEPES-KOH pH 7.5, 140 mM NaCl, 1 mM EDTA, 1% Triton X-100, 0.1% Na-Deoxycholate, proteases inhibitors (Roche)). The lysate was clarified by centrifugation at 15 000 rpm for 10 min. 20 µl of HA-coated agarose beads (Sigma) were added before incubation under slow agitation at 4°C for 4 hours. Beads were washed three times with co-IP buffer and resuspended in Laemmli buffer before Western blot analysis.

### Fluorescence microscopy

Cells were washed with PBS and mounted directly onto glass slides covered with polylysine (Sigma) in a drop of mounting solution (75% glycerol in PBS containing DAPI 0.05 µg/ml). Data were acquired using a Leica DM RXA microscope, equipped with a piezoelectric translator (PIFOC; PI) placed at the base of a 100× PlanApo N.A. 1.4 objective, and a 5 MHz Micromax 1300Y interline CCD camera (Roper Instruments). For the acquisition of Z-series, stacks of fluorescence images were collected automatically at 0.2 µm Z-distance (Metamorph software; Molecular Devices). Data were processed using Image J software.

### Yeast Two-hybrid

We used LexA-based two-hybrid technology (Clontech). *DRE2,* Ciapin1 and *TAH18* ORFs were amplified by PCR using oligos 214, 215, 313 and 314 (see Table 1), and cloned into Activating domain containing plasmid pB42 using *EcoR*I*-Xho*I. *TAH18* was amplified using oligos 216 and 217 and cloned into Binding domain containing plasmid pGilda using *EcoR*I*-Xho*I. Strain EGY48 was transformed with p8op-lacZ to create EGY48[p8op-lacZ]. EGY48[p8op-lacZ] was then co-transformed with the different fusion plasmids used in this study and clones were assayed for growth and blue colour onto GR and GRLX media, according to the manufacturer's instructions.

## References

[1] Chanet R, Heude M (2003). Characterization of mutations that are synthetic lethal with pol3-13, a mutated allele of DNA polymerase delta in Saccharomyces cerevisiae.. Curr Genet.

[2] Vachova L, Palkova Z (2005). Physiological regulation of yeast cell death in multicellular colonies is triggered by ammonia.. J Cell Biol.

[3] Ivanovska I, Hardwick JM (2005). Viruses activate a genetically conserved cell death pathway in a unicellular organism.. J Cell Biol.

[4] Madeo F, Frohlich E, Ligr M, Grey M, Sigrist SJ (1999). Oxygen stress: a regulator of apoptosis in yeast.. J Cell Biol.

[5] Ludovico P, Sousa MJ, Silva MT, Leao C, Corte-Real M (2001). Saccharomyces cerevisiae commits to a programmed cell death process in response to acetic acid.. Microbiology.

[6] Ludovico P, Sansonetty F, Silva MT, Corte-Real M (2003). Acetic acid induces a programmed cell death process in the food spoilage yeast Zygosaccharomyces bailii.. FEMS Yeast Res.

[7] Huh GH, Damsz B, Matsumoto TK, Reddy MP, Rus AM (2002). Salt causes ion disequilibrium-induced programmed cell death in yeast and plants.. Plant J.

[8] Wadskog I, Maldener C, Proksch A, Madeo F, Adler L (2004). Yeast lacking the SRO7/SOP1-encoded tumor suppressor homologue show increased susceptibility to apoptosis-like cell death on exposure to NaCl stress.. Mol Biol Cell.

[9] Del Carratore R, Della Croce C, Simili M, Taccini E, Scavuzzo M (2002). Cell cycle and morphological alterations as indicative of apoptosis promoted by UV irradiation in S. cerevisiae.. Mutat Res.

[10] Severin FF, Hyman AA (2002). Pheromone induces programmed cell death in S. cerevisiae.. Curr Biol.

[11] Madeo F, Frohlich E, Frohlich KU (1997). A yeast mutant showing diagnostic markers of early and late apoptosis.. J Cell Biol.

[12] Ye Y, Meyer HH, Rapoport TA (2003). Function of the p97-Ufd1-Npl4 complex in retrotranslocation from the ER to the cytosol: dual recognition of nonubiquitinated polypeptide segments and polyubiquitin chains.. J Cell Biol.

[13] Madeo F, Herker E, Maldener C, Wissing S, Lachelt S (2002). A caspase-related protease regulates apoptosis in yeast.. Mol Cell.

[14] Wissing S, Ludovico P, Herker E, Buttner S, Engelhardt SM (2004). An AIF orthologue regulates apoptosis in yeast.. J Cell Biol.

[15] Suzuki Y, Imai Y, Nakayama H, Takahashi K, Takio K (2001). A serine protease, HtrA2, is released from the mitochondria and interacts with XIAP, inducing cell death.. Mol Cell.

[16] Fahrenkrog B, Sauder U, Aebi U (2004). The S. cerevisiae HtrA-like protein Nma111p is a nuclear serine protease that mediates yeast apoptosis.. J Cell Sci.

[17] Manon S, Chaudhuri B, Guerin M (1997). Release of cytochrome c and decrease of cytochrome c oxidase in Bax-expressing yeast cells, and prevention of these effects by coexpression of Bcl-xL.. FEBS Lett.

[18] Ligr M, Madeo F, Frohlich E, Hilt W, Frohlich KU (1998). Mammalian Bax triggers apoptotic changes in yeast.. FEBS Lett.

[19] Pati D, Zhang N, Plon SE (2002). Linking sister chromatid cohesion and apoptosis: role of Rad21.. Mol Cell Biol.

[20] Chen F, Kamradt M, Mulcahy M, Byun Y, Xu H (2002). Caspase proteolysis of the cohesin component RAD21 promotes apoptosis.. J Biol Chem.

[21] Yang H, Ren Q, Zhang Z (2008). Cleavage of Mcd1 by Caspase-like Protease Esp1 Promotes Apoptosis in Budding Yeast.. Mol Biol Cell.

[22] Zhang Y, Lyver ER, Nakamaru-Ogiso E, Yoon H, Amutha B (2008). Dre2, a Conserved Eukaryotic Fe/S Cluster Protein, Functions in Cytosolic Fe/S Protein Biogenesis.. Mol Cell Biol.

[23] Shibayama H, Takai E, Matsumura I, Kouno M, Morii E (2004). Identification of a cytokine-induced antiapoptotic molecule anamorsin essential for definitive hematopoiesis.. J Exp Med.

[24] Simon M, Giot L, Faye G (1993). A random mutagenesis procedure: application to the POL3 gene of Saccharomyces cerevisiae.. Gene.

[25] Reid RJ, Fiorani P, Sugawara M, Bjornsti MA (1999). CDC45 and DPB11 are required for processive DNA replication and resistance to DNA topoisomerase I-mediated DNA damage.. Proc Natl Acad Sci U S A.

[26] Fiorani P, Reid RJ, Schepis A, Jacquiau HR, Guo H (2004). The deubiquitinating enzyme Doa4p protects cells from DNA topoisomerase I poisons.. J Biol Chem.

[27] Stewart L, Redinbo MR, Qiu X, Hol WG, Champoux JJ (1998). A model for the mechanism of human topoisomerase I.. Science.

[28] Pourquier P, Pommier Y (2001). Topoisomerase I-mediated DNA damage.. Adv Cancer Res.

[29] Burkart W, Jung T, Frasch G (1999). Damage pattern as a function of radiation quality and other factors.. C R Acad Sci III.

[30] Maris AF, Kern AL, Picada JN, Boccardi F, Brendel M (2000). Glutathione, but not transcription factor Yap1, is required for carbon source-dependent resistance to oxidative stress in Saccharomyces cerevisiae.. Curr Genet.

[31] Macierzynska E, Grzelak A, Bartosz G (2007). The effect of growth medium on the antioxidant defense of Saccharomyces cerevisiae.. Cell Mol Biol Lett.

[32] Meisinger C, Pfanner N, Truscott KN (2006). Isolation of yeast mitochondria.. Methods Mol Biol.

[33] Stridh H, Kimland M, Jones DP, Orrenius S, Hampton MB (1998). Cytochrome c release and caspase activation in hydrogen peroxide- and tributyltin-induced apoptosis.. FEBS Lett.

[34] Ludovico P, Rodrigues F, Almeida A, Silva MT, Barrientos A (2002). Cytochrome c release and mitochondria involvement in programmed cell death induced by acetic acid in Saccharomyces cerevisiae.. Mol Biol Cell.

[35] Dutta C, Henry HL (1990). Detection of hemoprotein peroxidase activity on polyvinylidene difluoride membrane.. Anal Biochem.

[36] Huh WK, Falvo JV, Gerke LC, Carroll AS, Howson RW (2003). Global analysis of protein localization in budding yeast.. Nature.

[37] Mumberg D, Muller R, Funk M (1995). Yeast vectors for the controlled expression of heterologous proteins in different genetic backgrounds.. Gene.

[38] Wolter KG, Hsu YT, Smith CL, Nechushtan A, Xi XG (1997). Movement of Bax from the cytosol to mitochondria during apoptosis.. J Cell Biol.

[39] Gross A, McDonnell JM, Korsmeyer SJ (1999). BCL-2 family members and the mitochondria in apoptosis.. Genes Dev.

[40] Feng Y, Ariza ME, Goulet AC, Shi J, Nelson MA (2005). Death-signal-induced relocalization of cyclin-dependent kinase 11 to mitochondria.. Biochem J.

[41] Luo X, Budihardjo I, Zou H, Slaughter C, Wang X (1998). Bid, a Bcl2 interacting protein, mediates cytochrome c release from mitochondria in response to activation of cell surface death receptors.. Cell.

[42] Gross A, Yin XM, Wang K, Wei MC, Jockel J (1999). Caspase cleaved BID targets mitochondria and is required for cytochrome c release, while BCL-XL prevents this release but not tumor necrosis factor-R1/Fas death.. J Biol Chem.

[43] Sawada M, Sun W, Hayes P, Leskov K, Boothman DA (2003). Ku70 suppresses the apoptotic translocation of Bax to mitochondria.. Nat Cell Biol.

[44] Lazazzera BA, Beinert H, Khoroshilova N, Kennedy MC, Kiley PJ (1996). DNA binding and dimerization of the Fe-S-containing FNR protein from Escherichia coli are regulated by oxygen.. J Biol Chem.

[45] Flint DH, Smyk-Randall E, Tuminello JF, Draczynska-Lusiak B, Brown OR (1993). The inactivation of dihydroxy-acid dehydratase in Escherichia coli treated with hyperbaric oxygen occurs because of the destruction of its Fe-S cluster, but the enzyme remains in the cell in a form that can be reactivated.. J Biol Chem.

[46] Flint DH, Tuminello JF, Emptage MH (1993). The inactivation of Fe-S cluster containing hydro-lyases by superoxide.. J Biol Chem.

[47] Collinson LP, Dawes IW (1992). Inducibility of the response of yeast cells to peroxide stress.. J Gen Microbiol.

[48] Lee J, Godon C, Lagniel G, Spector D, Garin J (1999). Yap1 and Skn7 control two specialized oxidative stress response regulons in yeast.. J Biol Chem.

[49] Ng CH, Tan SX, Perrone GG, Thorpe GW, Higgins VJ (2008). Adaptation to hydrogen peroxide in Saccharomyces cerevisiae: the role of NADPH-generating systems and the SKN7 transcription factor.. Free Radic Biol Med.

[50] Fiorani P, Bjornsti MA (2000). Mechanisms of DNA topoisomerase I-induced cell killing in the yeast Saccharomyces cerevisiae.. Ann N Y Acad Sci.

[51] Klinge S, Hirst J, Maman JD, Krude T, Pellegrini L (2007). An iron-sulfur domain of the eukaryotic primase is essential for RNA primer synthesis.. Nat Struct Mol Biol.

[52] Hausmann A, Aguilar Netz DJ, Balk J, Pierik AJ, Muhlenhoff U (2005). The eukaryotic P loop NTPase Nbp35: an essential component of the cytosolic and nuclear iron-sulfur protein assembly machinery.. Proc Natl Acad Sci U S A.

[53] Ben-Aroya S, Coombes C, Kwok T, O'Donnell KA, Boeke JD (2008). Toward a comprehensive temperature-sensitive mutant repository of the essential genes of Saccharomyces cerevisiae.. Mol Cell.

[54] Zhou YH, Zhang XP, Ebright RH (1991). Random mutagenesis of gene-sized DNA molecules by use of PCR with Taq DNA polymerase.. Nucleic Acids Res.

[55] Longtine MS, McKenzie A, Demarini DJ, Shah NG, Wach A (1998). Additional modules for versatile and economical PCR-based gene deletion and modification in Saccharomyces cerevisiae.. Yeast.

[56] Sikorski RS, Hieter P (1989). A system of shuttle vectors and yeast host strains designed for efficient manipulation of DNA in Saccharomyces cerevisiae.. Genetics.

[57] Foiani M, Marini F, Gamba D, Lucchini G, Plevani P (1994). The B subunit of the DNA polymerase alpha-primase complex in Saccharomyces cerevisiae executes an essential function at the initial stage of DNA replication.. Mol Cell Biol.

